# *Streptococcus Mutans* Membrane Vesicles Enhance *Candida albicans* Pathogenicity and Carbohydrate Metabolism

**DOI:** 10.3389/fcimb.2022.940602

**Published:** 2022-07-26

**Authors:** Ruixue Wu, Guxin Cui, Yina Cao, Wei Zhao, Huancai Lin

**Affiliations:** ^1^ Guangdong Provincial Key Laboratory of Stomatology, Sun Yat-sen University, Guangzhou, China; ^2^ Department of Pediatric Dentistry, Hospital of Stomatology, Guanghua School of Stomatology, Sun Yat-sen University, Guangzhou, China; ^3^ Department of Preventive Dentistry, Hospital of Stomatology, Guanghua School of Stomatology, Sun Yat-sen University, Guangzhou, China

**Keywords:** *Streptococcus mutans*, *Candida albicans*, membrane vesicles, cross-kingdom, dental caries

## Abstract

*Streptococcus mutans* and *Candida albicans*, as the most common bacterium and fungus in the oral cavity respectively, are considered microbiological risk markers of early childhood caries. *S. mutans* membrane vesicles (MVs) contain virulence proteins, which play roles in biofilm formation and disease progression. Our previous research found that *S. mutans* MVs harboring glucosyltransferases augment *C. albicans* biofilm formation by increasing exopolysaccharide production, but the specific impact of *S. mutans* MVs on *C. albicans* virulence and pathogenicity is still unknown. In the present study, we developed *C. albicans* biofilms on the surface of cover glass, hydroxyapatite discs and bovine dentin specimens. The results showed that *C. albicans* can better adhere to the tooth surface with the effect of *S. mutans* MVs. Meanwhile, we employed *C. albicans* biofilm-bovine dentin model to evaluate the influence of *S. mutans* MVs on *C. albicans* biofilm cariogenicity. In the *S. mutans* MV-treated group, the bovine dentin surface hardness loss was significantly increased and the surface morphology showed more dentin tubule exposure and broken dentin tubules. Subsequently, integrative proteomic and metabolomic approaches were used to identify the differentially expressed proteins and metabolites of *C. albicans* when cocultured with *S. mutans* MVs. The combination of proteomics and metabolomics analysis indicated that significantly regulated proteins and metabolites were involved in amino acid and carbohydrate metabolism. In summary, the results of the present study proved that *S. mutans* MVs increase bovine dentin demineralization provoked by *C. albicans* biofilms and enhance the protein and metabolite expression of *C. albicans* related to carbohydrate metabolism.

## Introduction

*Streptococcus mutans*, an important cariogenic bacterium in the oral cavity, is considered a microbiological risk marker of dental caries (Grier et al., 2021). *Candida albicans*, the most common fungus in the oral cavity, is both acidogenic and aciduric and in this regard has similar properties to *S. mutans* (Klinke et al., 2009; Pereira et al., 2018). A number of studies have reported that *C. albicans* is able to adhere to the surfaces of hydroxyapatite, dentin and cementum (Pereira et al., 2018; Thanh Nguyen et al., 2021). In addition, secreted aspartic proteases of *C. albicans* can degrade dentin collagen fibers and may play an important role in early childhood caries (ECC) (Li et al., 2014). Recent research found that *C. albicans* is frequently isolated in the oral mycobiome of early childhood caries and causes more severe dental caries (Falsetta et al., 2014; Cui et al., 2021; Alkhars et al., 2022). As the core resident of dental plaque biofilms, *C. albicans* interacts with various oral bacteria, such as *Streptococcus gordonii* and *S. mutans* (Bertolini and Dongari-Bagtzoglou, 2019). *C. albicans* may be a “keystone commensal” of plaque biofilms and can work synergistically with classic cariogenic bacteria (Pereira et al., 2018; Young et al., 2020).

The cross-kingdom interactions of *S. mutans* and *C. albicans* are considered to be associated with severe dental caries (Koo et al., 2018), which has become of increasing interest. *S. mutans* is involved in *C. albicans* virulence regulation in dental plaque biofilms. Mutanobactin and competence-stimulating peptide of *S. mutans* inhibit *C. albicans* hyphal formation (Joyner et al., 2010). However, *S. mutans* degrades sucrose into glucose and fructose, which provide for *C. albicans* catabolism in the sucrose environment (Falsetta et al., 2014). *S. mutans* antigen I/II and glucosyltransferase (Gtfs) secreted by *S. mutans* promote *C. albicans* adhesion and biofilm formation (Ellepola et al., 2017; Yang et al., 2018). *S. mutans* GtfB can bind to mannan on the cell surface of *C. albicans* and promote coadhesion and symbiotic biofilm development (Koo et al., 2018).

*S. mutans* membrane vesicles (MVs) contain proteins, extracellular DNA (eDNA) and other biologically active substances that play roles in cell-to-cell communication, biofilm formation and disease progression (Cao and Lin, 2021). The *S. mutans sfp*, *srtA* and TnSmu2 genes are involved in the formation and secretion of MVs (Morales-Aparicio et al., 2020; Wen et al., 2021). It has been demonstrated that *S. mutans* MVs are involved in cell wall synthesis, bacterial adhesion and biofilm formation (Cao et al., 2020; Morales-Aparicio et al., 2020). Moreover, they can promote the biofilm formation of *Streptococcus mitis*, *Streptococcus oralis*, *C. albicans* and other microorganisms in the oral cavity (Senpuku et al., 2019; Wu et al., 2020). Our previous research found that *S. mutans* MVs harboring glucosyltransferases augment *C. albicans* biofilm formation by increasing exopolysaccharide production (Cao et al., 2020; Wu et al., 2020). However, the specific impact of *S. mutans* MVs on *C. albicans* virulence and pathogenicity is still unknown.

In the present study, we employed a *C. albicans* biofilm-bovine dentin model to evaluate the effect of *S. mutans* MVs on *C. albicans* biofilm pathogenicity. An integrative proteomic and metabolomic approach was used to identify the differentially expressed proteins and metabolites of *C. albicans*. The present study will identify the specific influence of *S. mutans* MVs on *C. albicans* cariogenic ability.

## Materials and Methods

### Bacterial Strains And Culture Conditions

*S. mutans* UA159 (ATCC 700610) (Wu et al., 2020) and *C. albicans* SC5314 (ATCC MYA-2876) (Wu et al., 2020) were used in the present study. Brain heart infusion (BHI; Difco, Detroit, MI, United States) was employed to cultivate *S. mutans*, and Sabouraud’s dextrose broth (SDB, HKM, Guangzhou, China) was used to cultivate *C. albicans*. *C. albicans* biofilm development was achieved using- tryptone-yeast extract (TYE, OXOID, Hampshire, United Kingdom) medium supplemented with 1% sucrose. The method of cell culture was performed according to a previous study (Wu et al., 2020).

### Preparation of *S. mutans* MVs

For the preparation of MVs from *S. mutans*, *S. mutans* was grown in 500 mL BHI broth at 37 °C for 16 h. The culture was centrifuged at 6,000 × *g* for 15 min at 4 °C and at 10,000 × *g* for 15 min at 4 °C to remove cells and cell debris. The supernatants were removed and filtered through 0.22 μm filters (Millipore, MMAS, United States). Then, the supernatants were concentrated by a 100 kDa Amicon ultrafiltration system (Millipore, MMAS, United States). *S. mutans* MVs were harvested by centrifugation at 100,000 × *g* for 70 min at 4° C. The *S. mutans* MVs yield was quantified using a BCA assay (CWBIO, Beijing, China) as we previously described (Wu et al., 2020).

### Preparation of Bovine Dentin Specimens And Hydroxyapatite Discs

Bovine dentin specimens were prepared as we previously described (Wu et al., 2018). Briefly, dentin specimens (5 mm × 4 mm × 2 mm) were prepared from sound incisor bovine teeth that were freshly extracted. These teeth were devoid of stains, erosion and microcracks. Hydroxyapatite (HA) discs (Clarkson Chromatography Products, South Williamsport, PA, USA) 9.7 mm in diameter and 1.5 mm thick were used in this study. Dentin surfaces and HA discs were ground and polished by silicon carbide sandpapers (600-, 800-, 1500- and 2000-grit) in order to avoid the influence of HA discs surface roughness in *C. albicans* biofilm formation (Wang et al., 2020b; Le et al., 2022). After polishing, the dentin specimens and HA discs were sonicated in distilled water for 10 min to remove residual debris. Before the start of the experiment, the dentin specimens were disinfected with 75% alcohol for 12 h and sterilized by ultraviolet light for 4 h, and the HA discs were autoclaved at 121 °C for 15 min.

### *C. albicans* Biofilm Formation

*C. albicans* biofilms were cultured on the surfaces of cover glass, HA discs and dentin specimens to assess the effect of *S. mutans* MVs on *C. albicans* biofilm formation. *C. albicans* biofilms were developed as we previously described (Wu et al., 2020), with slight modification. First, all cover glass, HA discs and dentin specimens were preserved in sterile artificial saliva (ChangFeng Technology, Guangzhou, China) for 2 h at 37 °C. Then, artificial saliva was removed, and SDB containing *C. albicans* (~10^6^ CFU/mL) was added to culture plates and incubated at 37 °C for 90 min with a shaking speed of 90 rpm under aerobic conditions. Immediately afterward, sterile PBS was employed to wash unattached cells, and 1% sucrose TYE medium containing 40 μg/mL *S. mutans* MVs was added to culture plates to develop *C. albicans* biofilms at 37 °C under aerobic conditions for 24-96 h. The 1% sucrose TYE culture medium containing *S. mutans* MVs was replaced daily. As the control group, *C. albicans* biofilms were cultured with 1% sucrose TYE medium without *S. mutans* MVs. Dentin specimens mixed with 1% sucrose TYE medium containing 40 μg/mL *S. mutans* MVs without *C. albicans* inoculation was the blank control group. Analysis of *C. albicans* biofilms and dentin specimens by SEM.

Scanning electron microscopy (SEM) (Quanta 400F-FEI, Eindhoven, Netherlands) was employed to observe *C. albicans* biofilm and dentin specimen morphological characteristics (Wu et al., 2020). For *C. albicans* biofilms on cover glass, HA discs and dentin specimen analysis, the supernatants and unattached cells were removed by washing with sterile PBS three times. For dentin specimen surface observation, the attached *C. albicans* biofilm was removed by washing with deionized water. After fixation, dehydration and gold sputter-coating, the *C. albicans* biofilm was observed at 2,000 × magnification by SEM, and dentin specimens were observed at 4,000 × and 8,000 × magnification by SEM. The experiment was performed in 4 biological replicates.

### Measurement of Dentin Surface Microhardness and Roughness

*C. albicans* biofilms were developed on the dentin surface over 96 h, with culture medium replaced daily. Following biofilm development, the attached *C. albicans* biofilm was removed by washing with deionised water and dentin samples were stored in PBS at 4°C.

A Vikers microhardness tester (DuraScan-20, Struers, Germany) was employed to measure the surface microhardness of the dentin specimens. Three random points on each dentin specimen surface were subjected to measurement with a load of 0.2 HV for 15 s. The dentin surface hardness (SH) of each group (n = 8) was determined before and after *C. albicans* biofilm formation. Surface hardness loss (%SHL) is an indicator of demineralization, which was calculated as %SHL=(SH_1_-SH_2_)/SH_1_ × 100%, with SH_1_ being the SH of the dentin specimen before *C. albicans* biofilm formation, and SH_2_ being the SH of the dentin specimen after *C. albicans* biofilm formation (Sampaio et al., 2019). SH_1_ and SH_2_ were measured at three different defined regions of the sample, and the mean values were calculated. Confocal laser scanning microscopy (CLSM) (LSM700-Carl, Zeiss, Germany) was used to detect the surface roughness of the dentin specimens. Three measurement areas of 300 μm × 300 μm on each dentin specimen surface were randomly selected to obtain three-dimensional topography images and analyze the average roughness (Ra). The Ra of each group (n = 8) was determined before and after *C. albicans* biofilm formation, and the increase in the surface roughness of the dentin was calculated. Ra was measured at three different defined regions of the sample and the mean values were calculated.

### Sample Preparation for Proteome and Metabolome Analysis

*C. albicans* biofilms were developed on cover glass for 24 h, with supernatants and planktonic cells discarded following incubation. Biofilm samples were collected in PBS and washed by centrifugation at 12, 000 rpm for 5 min at 4°C. All samples were stored at -80°C for no longer than one month.

### TMT Labeling Proteomic Analyses

The TCA/acetone precipitation and SDT lysis method was employed to extract proteins. Protein quantitation was performed by a BCA assay (CWBIO, Beijing, China), and separation detected by SDS-PAGE. The *C. albicans* protein samples were digested according to the FASP procedure and labeled using TMT reagent according to the manufacturer’s instructions (Thermo Fisher Scientific). The subsequent procedures included peptide fractionation with reversed phase (RP) chromatography, mass spectrometry analysis, data analysis by Proteome Discoverer 2.2 software (Thermo Fisher Scientific), and bioinformatic analysis including Gene Ontology (GO) and KEGG Pathway annotations. Three biological replicates were prepared in each group. The final proteins that were deemed to be differentially expressed were screened by the following criteria: 1.2-fold changes (upregulation or downregulation) relative to the control group, and *P* value <0.05.

### Metabalomic Analysis

The metabolites of collected samples were extracted with 50% methanol buffer, and pooled quality control (QC) samples were prepared at the same time. An ultra-performance liquid chromatography (UPLC) system (Sciex, UK) and an ACQUITY UPLC T3 column (Waters, UK) were used for sample chromatographic separation and reversed phase separation. Metabolites eluted from the column were detected by a high-resolution tandem mass spectrometer (TripleTOF5600plus, Sciex, UK). The mass spectrometry data were acquired in IDA mode. The quality of the acquired LC–MS data was analyzed by XCMS software. MetaX software was employed for metabolite identification, and quantification, and then the differential metabolites were screened. Metabolites were annotated through the open access databases, KEGG and HMDB. Six biological replicates were prepared in each group.

### Integrated Analysis of Metabolomics and Proteomics

The KEGG pathway database (http://www.genome.jp/kegg) and the Gene Ontology database (ftp://ftp.ncbi.nih.gov/gene/DATA/gene2go.gz) were employed to analyze the significantly altered canonical pathways and molecular networks of differentially expressed metabolites and proteins. The final proteins and metabolites that were deemed to be differentially expressed were screened by the following criteria: proteins, 1.2-fold changes (upregulation or downregulation) relative to the control group, and *P* value <0.05; metabolites, 2.0-fold changes (upregulation or downregulation) relative to the control group, and *P* value <0.05.

### Statistical Analysis

Statistical analysis was performed using SPSS 20.0. Significant differences between two groups were analyzed by unpaired *t*-test and one-way ANOVA combined with a Student-Newman-Keuls (SNK) *post hoc* test. *P <* 0.05 was considered significant. Each assay was carried out as at least three biological replicates and three technical replicates.

## Results

### *S. mutans* MVs Promote *C. albicans*Biofilm Development

**Figure 1:**
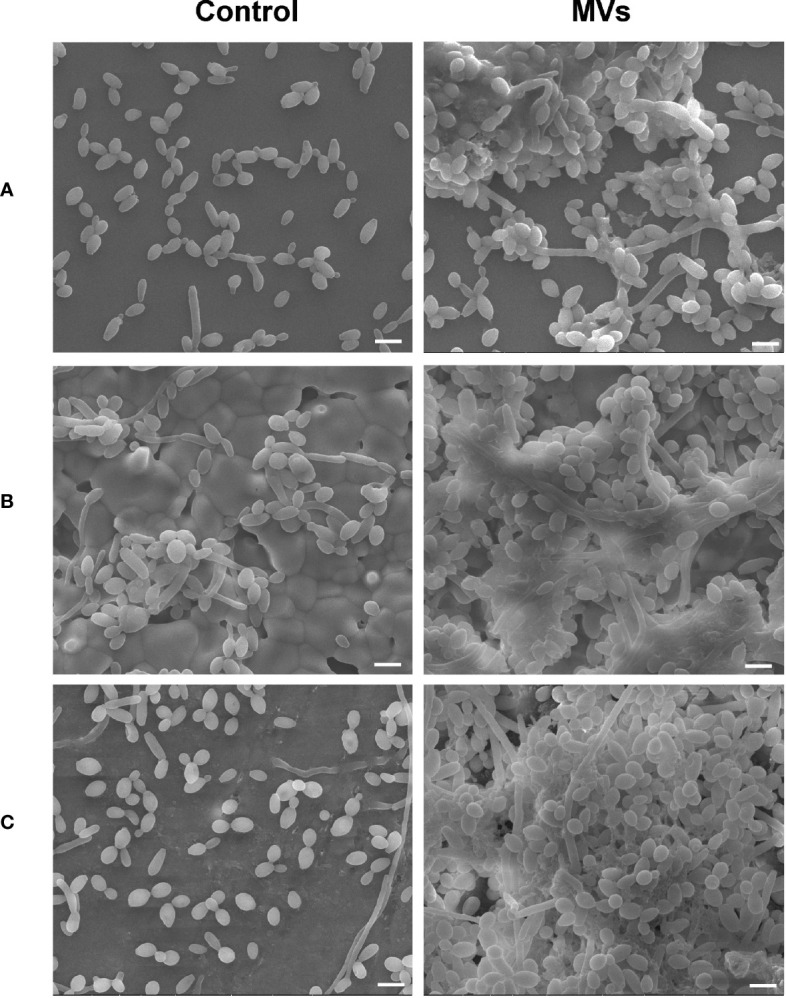
The effect of *S. mutans* MVs on *C. albicans* biofilm formation. *C. albicans* biofilms were developed on **(A)** cover glasses, **(B)** hydroxyapatite discs, and **(C)** bovine dentin specimens. Scale bar, 5 μm.

The *S. mutans* MVs used for this study were isolated by ultracentrifugation and characterized as previously described (Wu et al., 2020). SEM images showed that *S. mutans* MVs enhanced *C. albicans* clustering and biofilm formation on the surfaces of cover glass (**Figure 1A**), hydroxyapatite discs (**Figure 1B**) and bovine dentin specimens (**Figure 1C**). In the *S. mutans* MV-treated group, there was biofilm extracellular matrix formation, and the biofilm structure was three-dimensional (**Figure 1**).

### Effect of *S. mutans* MVs on *C. albicans* Biofilm Cariogenic Ability

**Figure 2:**
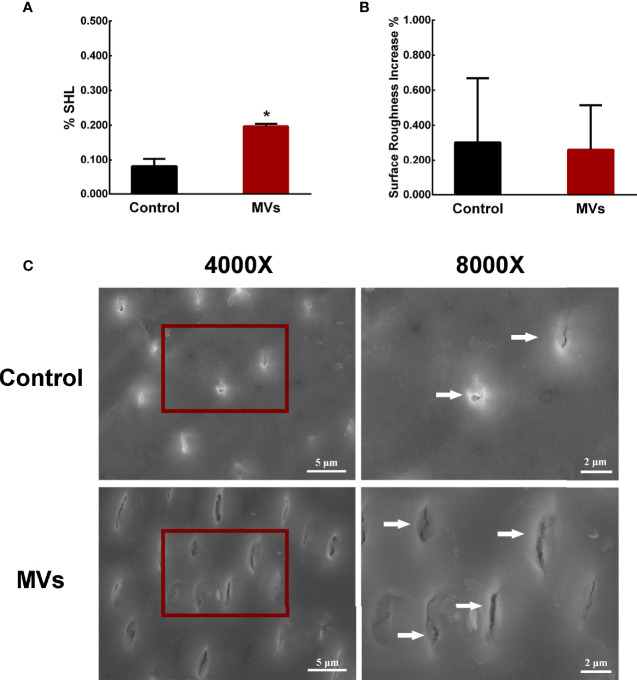
*S. mutans* MVs promote *C. albicans* biofilm formation to induce bovine dentin demineralization. **(A)** Dentine surface hardness loss (%SHL) (n = 8). **(B)** Dentine surface roughness increase (n = 8). **(C)** SEM images of dentin surfaces. Each field of view was magnified 4,000× and 8,000×. The red boxes indicate the magnified viewing area. The white arrows show the exposed dentin tubules. The data are presented as the means ± SD, *P < 0.05 vs control group.

A *C. albicans*-bovine dentin biofilm model was employed to evaluate the effect of *S. mutans* MVs on *C. albicans* biofilm cariogenic ability. The dentin surface hardness loss of the *S. mutans* MV-treated group was 19.66%, which was significantly different from that of the control group (**Figure 2A**, *P* < 0.05). The increase in dentin surface roughness between the *S. mutans* MV-treated group and the control group was not significantly different (**Figure 2B**, *P* > 0.05). Compared to the control group and blank control group, the dentin surface morphology of the *S. mutans* MV-treated group showed more dentin tubules exposure and broken dentin tubule (**Figure 2C**, **Supplementary Figure 1**).

### Effect of *S. mutans* MVs on *C. albicans* Based On Proteomic Analyses

**Figure 3:**
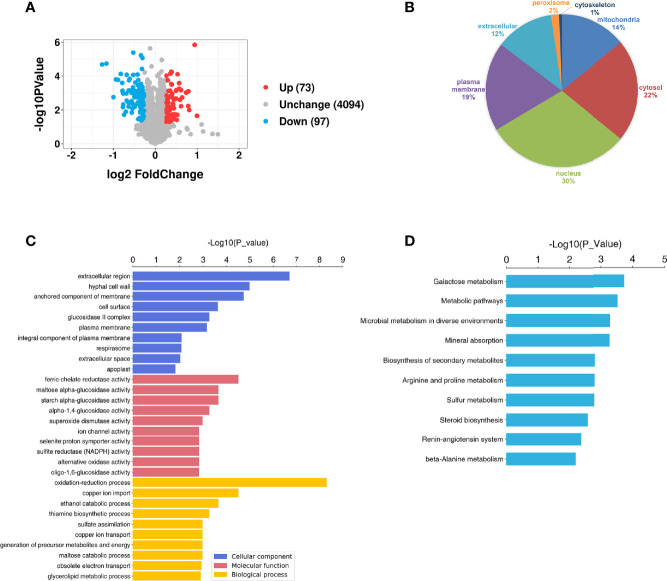
Proteomic analysis of *C. albicans.* **(A)** Volcano plot of differentially expressed *C. albicans* proteins. The red spots represent 73 proteins that were upregulated in the *S. mutans* MV group, and the blue spots represent 97 proteins that were downregulated in the *S. mutans* MV group (adjusted P < 0.05, fold change > 1.2). **(B)** Subcellular localization of proteins. **(C)** GO enrichment analysis. **(D)** KEGG enrichment analysis.

A total of 4345 proteins were identified in the *S. mutans* MV-treated and control groups by tandem mass tag (TMT) (**Supplementary Table 1**). For proteins of the *S. mutans* MV-treated group 1.2-fold changes (upregulation or downregulation) relative to the control group, and a *P* value <0.05 were deemed to be significant. The expression levels of 170 proteins were significantly changed as a result of *C. albicans* being treated with *S. mutans* MVs. The expression levels of 73 proteins were upregulated and 97 proteins were downregulated (**Figure 3A**). Principal component analysis (PCA) showed that the *S. mutans* MV-treated and control groups were clearly separated (**Supplementary Figure 2**). These results indicated that *S. mutans* MV significantly influenced the protein expression of *C. albicans*.

WoLF PSORT was employed to analyze the subcellular localization of proteins. The results showed that most of the significantly changed proteins were located in the nucleus and cytosol (**Figure 3B**). To identify the biological functions of significantly changed *C. albicans* proteins, GO enrichment analysis was performed (**Figure 3C**). The significantly changed proteins were mainly involved in biological processes such as oxidation–reduction processes and cellular components such as extracellular regions. KEGG analysis showed that the significantly changed proteins were mainly involved in galactose metabolism, which was similar to our previous findings that *S. mutans* MVs harboring Gtfs are involved in exopolysaccharide production in *C. albicans* biofilms (Wu et al., 2020). In addition, metabolic pathways, microbial metabolism in diverse environments and mineral absorption were also enriched (**Figure 3D**).

### Metabolomic Analysis of the Effect of *S. mutans* MVs on *C. albicans*

**Figure 4:**
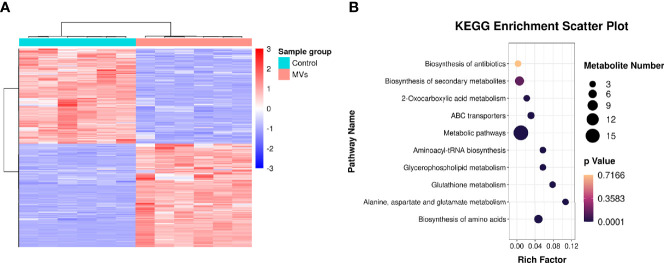
Metabonomic analysis of *C. albicans*. **(A)** Hierarchical clustering of *C. albicans* metabonomics. **(B)** KEGG enrichment analysis.

In total, 15,379 metabolites were identified in the *S. mutans* MV-treated and control groups (**Supplementary Table 2**). Metabolites of the *S. mutans* MV-treated group with 2.0-fold changes (upregulation or downregulation) relative to the control group and a *P* value <0.05 were deemed to be significantly changed. The expression levels of 937 metabolites were upregulated, and those of 857 metabolites were downregulated (**Supplementary Figure 3**). Quantitative metabolomic data of *C. albicans* samples were used for hierarchical clustering (**Figure 4A**). To identify the biological functions of significantly changed *C. albicans* metabolites under *S. mutans* MV treatment, KEGG enrichment analysis was performed (**Figure 4B**). The significantly regulated metabolites were mainly involved in metabolic pathways, which was consistent with the proteomics results. In addition, biosynthesis of amino acids, alanine, aspartate and glutamate metabolism, and glutathione metabolism were significantly enriched in regulated metabolites. These results indicated complex metabolic regulation in *S. mutans* MV-treated *C. albicans*.

### Integrated Analysis of Proteome and Metabolome Data in *C. albicans*

**Figure 5:**
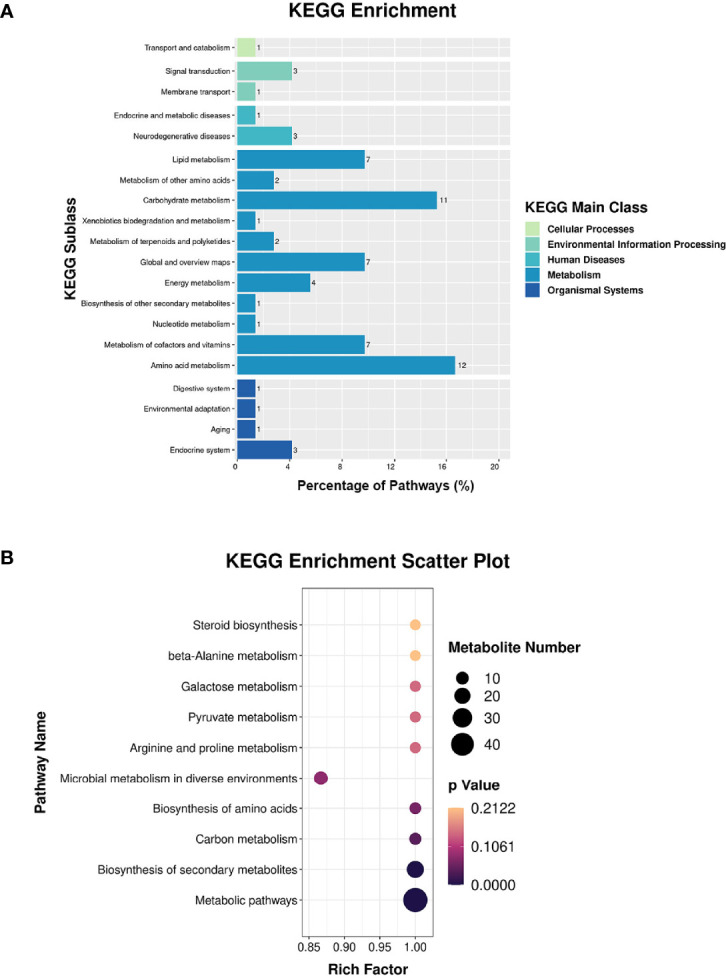
Integrated analysis of the proteome and the metabolome. **(A)** KEGG enrichment analysis. **(B)** Top-10 KEGG pathways involved in both proteomic and metabolic pathways.

Proteomic and metabolomic data were integrated through the same KEGG pathway, and proteins and metabolites involved in significant changes in the same biological process were identified. Most of the proteins and metabolites were involved in metabolic processes, including amino acid metabolism and carbohydrate metabolism, consistent with the proteomic and metabolomic results (**Figure 5A**). We further performed enrichment analysis of the KEGG pathways, and the top 10 KEGG pathways were identified (**Figure 5B**), which included metabolic pathways, biosynthesis of secondary metabolites, carbon metabolism, and biosynthesis of amino acids.

## Discussion

*C. albicans* biofilm consists of different morphological types of cells (yeast, pseudohyphal, and hyphal) and extracellular polymeric substances. *C. albicans* biofilm production is an important virulence factor of *C. albicans* (Lohse et al., 2018). Our study showed that *S. mutans* MVs promote *C. albicans* biofilm formation on the surface of cover glass, hydroxyapatite discs and bovine dentin specimens. This indicates that *C. albicans* can better adhere to tooth surfaces with the effect of *S. mutans* MVs. Experimental studies have reported that *C. albicans* enhances the severity of dental caries because of its synergistic action with classical cariogenic bacteria (Falsetta et al., 2014; Pereira et al., 2018; Kim et al., 2020). Coinfection with *S. mutans* and *C. albicans* increases the cariogenic potential of dental plaque biofilms, leading to aggressive carious lesions *in vitro* and *in vivo* (Falsetta et al., 2014; Sampaio et al., 2019). *C. albicans* colonization on the tooth root surface can disrupt the balance of dental plaque microbial ecology and enhance the cariogenic ability of dental plaque, leading to the severity of tooth demineralization and carious lesions (Du et al., 2021). However, the cariogenic ability of *C. albicans* biofilms with the influence of *S. mutans* MVs has not yet been evaluated.

To further investigate the effect of *S. mutans* MVs on *C. albicans* biofilm cariogenic potential, a *C. albicans* biofilm-bovine dentin model was employed according to Sampaio et al.’ experimental method (Sampaio et al., 2019). *S. mutans* MVs have no significant effect on the pH of the *C. albicans* biofilm culture medium supernatants, which was reported by our previous study (Wu et al., 2020). Enamel demineralization is mainly due to the acid production of plaque biofilms (Wu et al., 2018), and *C. albicans* is usually detected in dentin tubules (Klinke et al., 2011). Moreover, bovine teeth have structural composition similar to that of human teeth and are easy to access (Mellberg, 1992). Therefore, bovine dentin specimens were employed in this study. According to the results of bovine dentin surface hardness loss, the dentin surface hardness loss of the *S. mutans* MV-treated group was 19.66%, which was significantly higher than that of the control group. In the control group, *C. albicans* biofilms alone did not decrease bovine dentin hardness, which was consistent with previous research on *C. albicans* biofilms on dentin demineralization (Sampaio et al., 2019). The increase in dentin surface roughness between the *S. mutans* MV-treated group and the control group was not significant different. This result may be due to the adhesion of *C. albicans* on the bovine dentin surface influencing the surface roughness; the detection of surface roughness by CLSM can be easily affected by the environment, which could have led to the lack of a significant difference between the two groups. SEM images showed that the bovine dentin surface morphology of the *S. mutans* MV-treated group had more dentin tubule exposure and broken dentin tubules, indicating the erosion of peritubular dentin and consistent with previous research reports (Poggio et al., 2013; Su et al., 2021). Altogether, these findings provide evidence that *S. mutans* MVs increase bovine dentin demineralization provoked by *C. albicans* biofilms.

Proteomics analysis showed that a total of 4345 proteins were identified in the *S. mutans* MV-treated and control groups, similar to the findings of previous research on *C. albicans* proteomics detection (Wang et al., 2020a). The significantly changed proteins were mainly involved in biological processes involving oxidation–reduction processes and cellular components, including extracellular regions. Meanwhile, significantly changed proteins were mainly related to galactose metabolism, and metabolic pathways and microbial metabolism were also enriched. Biological processes and metabolic pathways are essential for *C. albicans* biofilm formation (Munusamy et al., 2021). In the proteomics analysis of *S. mutans*-*C. albicans* mixed-species biofilms, *C. albicans* proteins involved in carbohydrate metabolism and cell wall components such as mannan and glucan were upregulated (Ellepola et al., 2019). Metabolomic analysis demonstrated enhanced expression of *C. albicans* metabolites related to metabolic pathways when cocultured with *S. mutans* MVs, including biosynthesis of amino acids, alanine, aspartate and glutamate metabolism, and glutathione metabolism, consistent with the proteomic results. The combination of proteomics and metabolomics analysis indicated that significantly regulated proteins and metabolites were involved in amino acid and carbohydrate metabolism. Taken together, *S. mutans* MVs enhance the expression of proteins and metabolites of *C. albicans* related to carbohydrate metabolism.

*S. mutans* MVs carry various virulence proteins (Senpuku et al., 2019; Cao and Lin, 2021). Our previous study found that *S. mutans* MVs contain metabolic enzymes such as Gtfs, Gbps and DexA, which are related to carbohydrate metabolism (Cao et al., 2020). Gfts are critical virulence factors of *S. mutans* that participate in sucrose metabolism and mediate sucrose-dependent adhesion, increasing the colonization of oral microorganisms (Loesche, 1986; Zhang et al., 2021). Gtfs also play a functional role in the cross-kingdom interactions of *S. mutans* and *C. albicans*. In the sucrose environment, *S. mutans* secretes Gtfs and breaks down sucrose into glucose and fructose, and *C. albicans* can utilize monosaccharides efficiently (Ellepola et al., 2017). *S. mutans* GtfB binds to the cell surface of *C. albicans* and promotes *C. albicans* cell accumulation (Ellepola et al., 2017; Koo et al., 2018). Our previous study also demonstrated that *S. mutans* MVs harboring Gtfs promote exopolysaccharide production in *C. albicans* biofilms, and *C. albicans* genes correlated with mannan and glucan synthesis were upregulated by the effect of Gtfs in *S. mutans* MVs (Wu et al., 2020). Therefore, the reason why *S. mutans* MVs enhance *C. albicans* carbohydrate metabolism may be due to the effect of metabolic enzymes such as Gtfs carried by *S. mutans* MVs.

Carbohydrate metabolism can influence *C. albicans* pathogenicity (Vieira et al., 2010; Ene et al., 2014). The competitive consumption of glucose by *C. albicans* and macrophagocytes disrupts the glucose homeostasis of the host, which results in rapid macrophagocyte death (Tucey et al., 2018). *C. albicans* can escape from macrophage attack with the recovery of glycolysis ability (Ries et al., 2018). Moreover, recent research found that the carbohydrate metabolism of *C. albicans* biofilms is correlated with dental caries. *S. mutans* and *C. albicans* are considered microbiological risk markers of early childhood caries (Cui et al., 2021; Grier et al., 2021; Alkhars et al., 2022). Research found that *C. albicans* genes and proteins related to sugar transportation, pyruvate breakdown and the glyoxylate cycle were upregulated in *S. mutans*-*C. albicans* mixed-species biofilms (Ellepola et al., 2019). The transcriptomic analysis of dental plaque from the tooth root surface found that *C. albicans* genes associated with metabolic activity and glucose transportation were significantly enhanced in the dental caries group compared to the caries-free group (Ev et al., 2020). Our results also demonstrate that *S. mutans* MVs promote *C. albicans* carbohydrate metabolism and dentin demineralization provoked by *C. albicans* biofilms. Therefore, *C. albicans* promotes the occurrence and development of dental caries may be through the regulation of carbohydrate metabolism.

In summary, our present study showed that *S. mutans* MVs promoted *C. albicans* biofilm formation on the surfaces of cover glass, hydroxyapatite discs and bovine dentin specimens and increased bovine dentin demineralization provoked by *C. albicans* biofilms. Meanwhile, *S. mutans* MVs increased the protein and metabolite expression of *C. albicans* related to carbohydrate metabolism. Altogether, these results increase our understanding of *S. mutans* MVs on *C. albicans* virulence and pathogenicity. However, the effect of *S. mutans* MVs on *C. albicans* in dental plaque biofilms *in vitro* and *in vivo* needs to be confirmed, and the mechanism by which carbohydrate metabolism influences *C. albicans* cariogenic ability is complex and remains unclear. Further studies are needed to close the gap in knowledge of the contribution of *S. mutans* MVs to *C. albicans* virulence and pathogenicity.

## Data Availability Statement

The original contributions presented in the study are publicly available. The proteomic data have been deposited into the iProX database https://www.iprox.cn/page/DSV021.html;?url=16521827236345c1p password: cN3M), and metabolic data is uploaded to the metabolights database (MTBLS5048 www.ebi.ac.uk/metabolights/MTBLS5048).

## Author Contributions

RW and HL designed the research. RW executed the experiments and analyzed the data. GC and YC provided technical and theoretical support. RW, WZ and HL co-wrote and revised the manuscript. All authors read and approved the submitted versions.

## Funding

This work was supported by the National Natural Science Foundation of China (No. 81970928).

## Conflict of Interest

The authors declare that the research was conducted in the absence of any commercial or financial relationships that could be construed as a potential conflict of interest.

## Publisher’s Note

All claims expressed in this article are solely those of the authors and do not necessarily represent those of their affiliated organizations, or those of the publisher, the editors and the reviewers. Any product that may be evaluated in this article, or claim that may be made by its manufacturer, is not guaranteed or endorsed by the publisher.
