# Supplementary material for: Streptococcus Mutans Membrane Vesicles Enhance Candida albicans Pathogenicity and Carbohydrate Metabolism
Source: Front Cell Infect Microbiol. 2022 Jul 26;12:940602. doi: 10.3389/fcimb.2022.940602 (PMC9361861; doi:10.3389/fcimb.2022.940602)
Supplement: Supplementary file 1 [file DataSheet_1.docx]

Supplementary Material

# Supplementary Figures and Tables

## Supplementary Figures


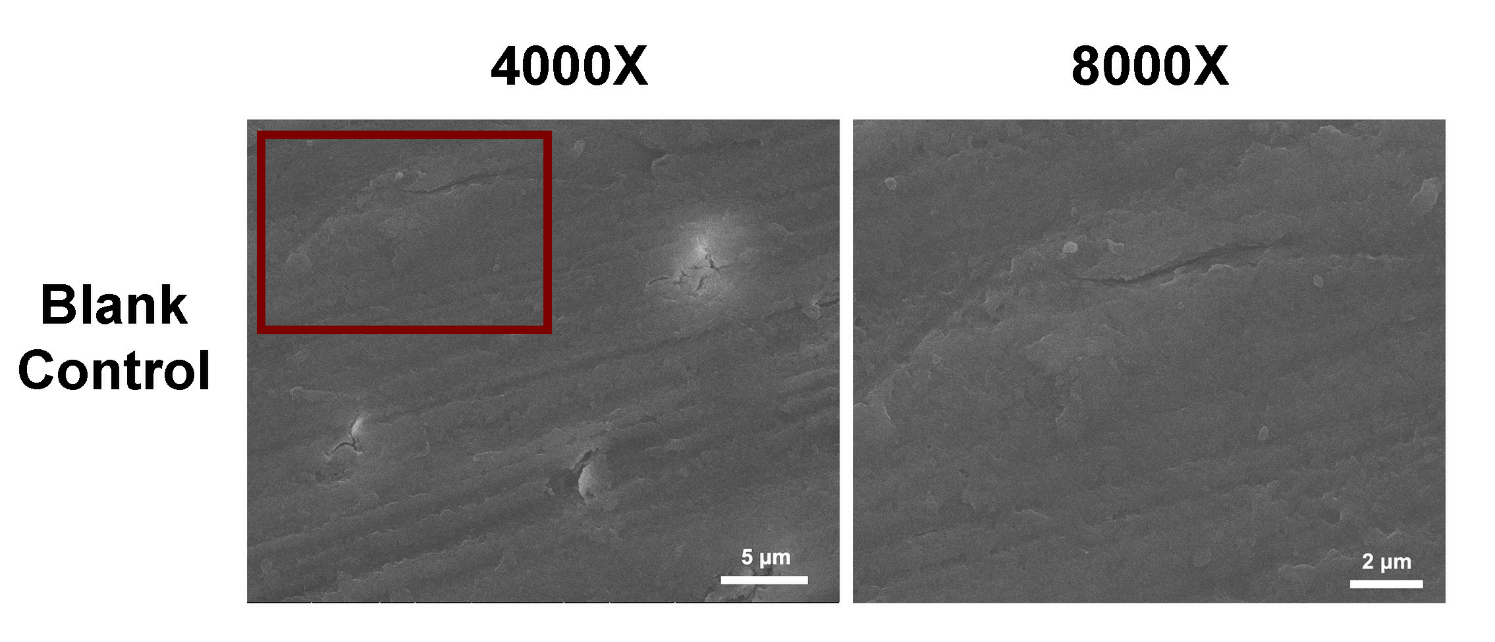
**Supplementary Figure 1.** SEM images of dentin surfaces of blank control group. Each field of view was magnified 4,000× and 8,000×. The red boxes indicate the magnified viewing area.
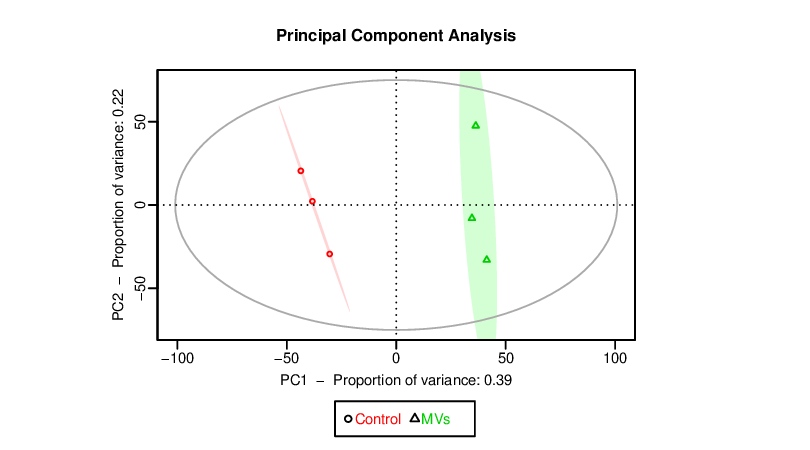


**Supplementary Figure 2.** Principal component analysis of *C. albicans* proteins.


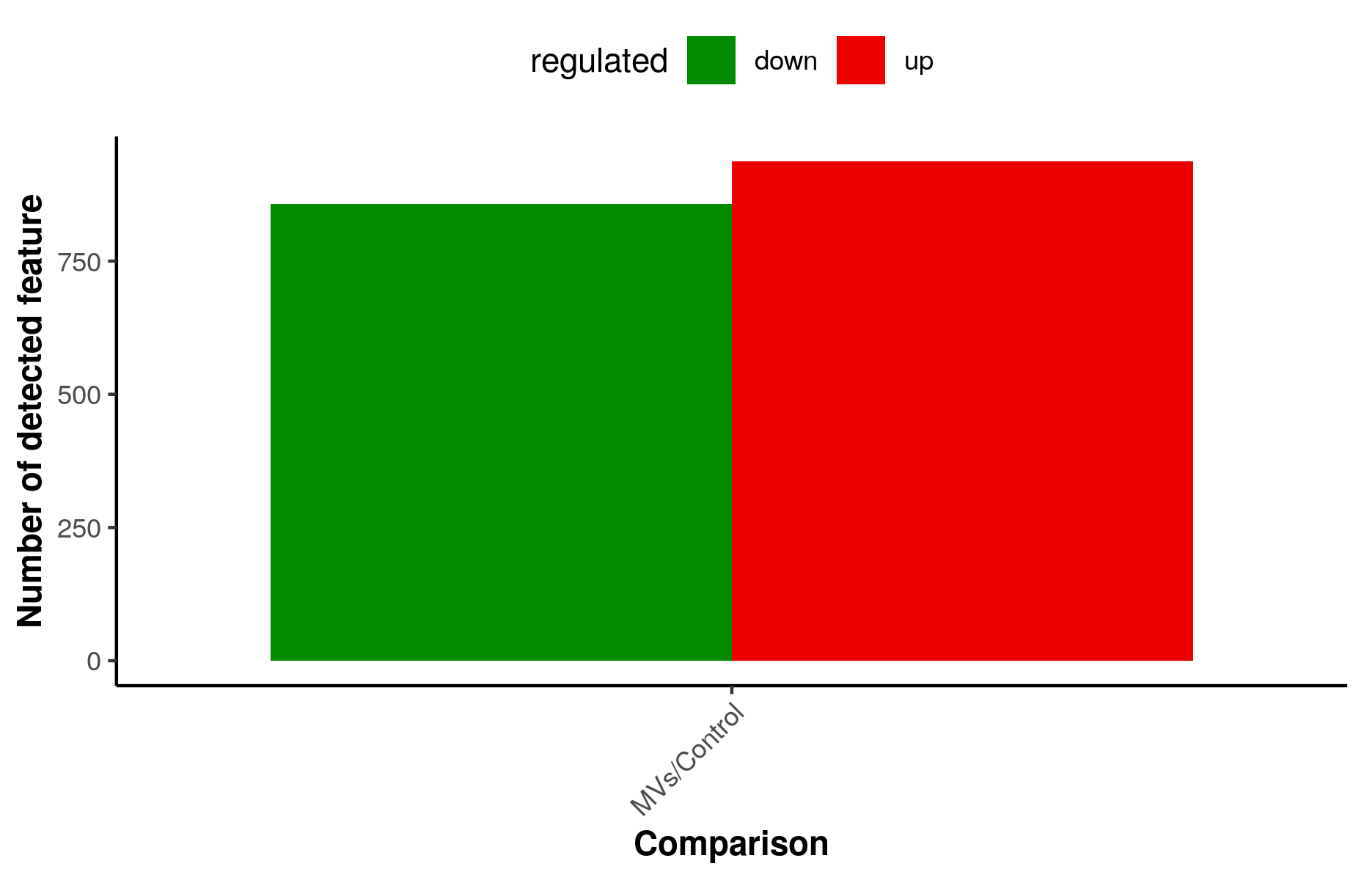


**Supplementary Figure 3** Bar chart of significant different *C. albicans* metabolites.

## Supplementary Tables

**Supplementary Table 1** *C. albicans* protein identification list.

**Supplementary Table 2** *C. albicans* metabolites identification list.

The proteomic raw data have been deposited into the iProX database

<https://www.iprox.cn/page/DSV021.html;?url=16521827236345c1p>

password: cN3M

The metabolites data is uploading to the metabolights database (MTBLS5048  [www.ebi.ac.uk/metabolights/MTBLS5048](http://www.ebi.ac.uk/metabolights/MTBLS5048)).
